# Supplementary material for: Wealth-based inequity in full child vaccination coverage: An experience from Mali, Bangladesh, and South Africa. A multilevel poison regression
Source: PLoS One. 2023 Dec 20;18(12):e0293522. doi: 10.1371/journal.pone.0293522 (PMC10732374; doi:10.1371/journal.pone.0293522)
Supplement: S1 Table — (DOCX) [file pone.0293522.s001.docx]

**S1 Table: The multilevel poison regression in full child vaccination and Equity Parameters in Mali, Bangladesh, and South Africa.**

**Mali**

| **Variables** | **Category** | **RR** | **Std. Err.** | **z** | **P>z** | **[95% Conf.**  **Interval]** | |
| --- | --- | --- | --- | --- | --- | --- | --- |
|  |  |  |  |  |  |  |  |
| Sex of the child | Male |  |  |  |  |  |  |
|  | Female | 1.04 | 0.05 | 0.76 | 0.45 | 0.94 | 1.15 |
| Age of the mother |  | 1.03 | 0.01 | 4.42 | 0.00 | 1.01 | 1.04 |
| Number of children |  | 0.95 | 0.02 | -2.91 | 0.00 | 0.91 | 0.98 |
| ANC | Less than 4 |  |  |  |  |  |  |
|  | 4 or more | 1.20 | 0.07 | 3.28 | 0.00 | 1.08 | 1.34 |
| Mother's education | No education |  |  |  |  |  |  |
|  | Primary | 1.05 | 0.08 | 0.58 | 0.56 | 0.89 | 1.23 |
|  | Secondary | 1.13 | 0.09 | 1.50 | 0.13 | 0.96 | 1.32 |
|  | Higher | 1.11 | 0.22 | 0.55 | 0.58 | 0.76 | 1.63 |
| Occupation | Not working |  |  |  |  |  |  |
|  | Working | 1.19 | 0.07 | 3.10 | 0.00 | 1.07 | 1.33 |
| Wealth quintile | Poorest | 0.70 | 0.10 | -2.62 | 0.01 | 0.54 | 0.91 |
|  | Poorer | 0.79 | 0.10 | -1.76 | 0.08 | 0.61 | 1.03 |
|  | Middle | 0.86 | 0.11 | -1.24 | 0.21 | 0.67 | 1.09 |
|  | Richer | 0.74 | 0.08 | -2.87 | 0.00 | 0.61 | 0.91 |
|  | Richest |  |  |  |  |  |  |
| Frequency of listening radio | Not at all |  |  |  |  |  |  |
|  | Less than once a week | 0.99 | 0.08 | -0.08 | 0.94 | 0.85 | 1.16 |
|  | At least once a week | 0.95 | 0.06 | -0.77 | 0.44 | 0.83 | 1.08 |
| Frequency of watching television | Not at all |  |  |  |  |  |  |
|  | Less than once a week | 1.19 | 0.09 | 2.24 | 0.03 | 1.02 | 1.38 |
|  | At least once a week | 1.21 | 0.09 | 2.59 | 0.01 | 1.05 | 1.39 |
| Distance to health facility | Big problem |  |  |  |  |  |  |
|  | Not a big problem | 1.04 | 0.06 | 0.63 | 0.53 | 0.92 | 1.17 |
| Place of residence | Urban |  |  |  |  |  |  |
|  | Rural | 1.31 | 0.14 | 2.59 | 0.01 | 1.07 | 1.61 |
|  | _cons | 0.11 | 0.02 | -12.44 | 0.00 | 0.08 | 0.16 |
|  |  |  |  |  |  |  |  |
| Cluster | v001 |  |  |  |  |  |  |
|  | var(_cons) | 0.13 | 0.03 |  |  | 0.08 | 0.20 |

**Bangladesh**

| **Variables** | **Category** | **RR** | **Std.Err** | **z** | **P>z** | **[95% Conf. Interval]** | |
| --- | --- | --- | --- | --- | --- | --- | --- |
|  |  |  |  |  |  |  |  |
| Sex of the child | Male |  |  |  |  |  |  |
|  | Female | 1.01 | 0.04 | 0.38 | 0.70 | 0.95 | 1.09 |
| Age of the mother |  | 1.02 | 0.00 | 4.82 | 0.00 | 1.01 | 1.03 |
| Number of children |  | 0.93 | 0.02 | -3.04 | 0.00 | 0.89 | 0.97 |
| ANC | Less than 4 |  |  |  |  |  |  |
|  | 4 or more | 1.10 | 0.04 | 2.54 | 0.01 | 1.02 | 1.19 |
| Mother's education | No education |  |  |  |  |  |  |
|  | Primary | 1.06 | 0.09 | 0.66 | 0.51 | 0.90 | 1.24 |
|  | Secondary | 1.14 | 0.09 | 1.55 | 0.12 | 0.97 | 1.34 |
|  | Higher | 1.06 | 0.10 | 0.60 | 0.55 | 0.88 | 1.27 |
| Occupation | Not working |  |  |  |  |  |  |
|  | Working | 1.13 | 0.04 | 3.21 | 0.00 | 1.05 | 1.22 |
| Wealth quintile | Poorest | 0.99 | 0.07 | -0.19 | 0.85 | 0.85 | 1.14 |
|  | Poorer | 0.96 | 0.07 | -0.54 | 0.59 | 0.84 | 1.10 |
|  | Middle | 0.92 | 0.06 | -1.37 | 0.17 | 0.81 | 1.04 |
|  | Richer | 0.94 | 0.06 | -1.13 | 0.26 | 0.83 | 1.05 |
|  | Richest |  |  |  |  |  |  |
| Frequency of listening radio | Not at all |  |  |  |  |  |  |
|  | Less than once a week | 1.02 | 0.10 | 0.24 | 0.81 | 0.85 | 1.23 |
|  | At least once a week | 1.00 | 0.12 | 0.03 | 0.98 | 0.79 | 1.27 |
| Frequency of watching television | Not at all |  |  |  |  |  |  |
|  | Less than once a week | 1.03 | 0.07 | 0.45 | 0.65 | 0.90 | 1.18 |
|  | At least once a week | 1.07 | 0.05 | 1.46 | 0.15 | 0.98 | 1.17 |
| Distance to health facility | Big problem |  |  |  |  |  |  |
|  | Not a big problem | 1.03 | 0.04 | 0.82 | 0.41 | 0.96 | 1.11 |
| Place of residence | Urban |  |  |  |  |  |  |
|  | Rural | 1.06 | 0.04 | 1.30 | 0.19 | 0.97 | 1.15 |
|  | _cons | 0.32 | 0.05 | -7.77 | 0.00 | 0.24 | 0.43 |
| Cluster | v001 |  |  |  |  |  |  |
|  | var(_cons) | 3.20E-108 | 1.67E-73 |  |  | . | . |

**South Africa**

| **Variables** | **Category** | **RR** | **Std.Err** | **z** | **P>z** | **[95% Conf. Interval]** | |
| --- | --- | --- | --- | --- | --- | --- | --- |
|  |  |  |  |  |  |  |  |
| Sex of the child | Male |  |  |  |  |  |  |
|  | Female | 1.00 | 0.09 | 0.03 | 0.98 | 0.83 | 1.20 |
| Age of the mother |  | 1.02 | 0.01 | 2.47 | 0.01 | 1.01 | 1.04 |
| Number of children |  | 0.92 | 0.05 | -1.61 | 0.11 | 0.84 | 1.02 |
| ANC | Less than 4 |  |  |  |  |  |  |
|  | 4 or more | 1.19 | 0.15 | 1.33 | 0.19 | 0.92 | 1.53 |
| Mother's education | No education |  |  |  |  |  |  |
|  | Primary | 0.73 | 0.27 | -0.86 | 0.39 | 0.35 | 1.51 |
|  | Secondary | 0.86 | 0.30 | -0.45 | 0.66 | 0.43 | 1.69 |
|  | Higher | 0.87 | 0.34 | -0.35 | 0.73 | 0.41 | 1.87 |
| Occupation | Not working |  |  |  |  |  |  |
|  | Working | 0.76 | 0.09 | -2.33 | 0.02 | 0.61 | 0.96 |
| Wealth quintile | Poorest | 1.66 | 0.39 | 2.17 | 0.03 | 1.05 | 2.64 |
|  | Poorer | 1.33 | 0.29 | 1.33 | 0.19 | 0.87 | 2.04 |
|  | Middle | 1.23 | 0.25 | 1.00 | 0.32 | 0.82 | 1.84 |
|  | Richer | 1.16 | 0.24 | 0.71 | 0.48 | 0.77 | 1.74 |
|  | Richest |  |  |  |  |  |  |
| Frequency of listening radio | Not at all |  |  |  |  |  |  |
|  | Less than once a week | 1.00 | 0.15 | -0.02 | 0.98 | 0.74 | 1.34 |
|  | At least once a week | 1.14 | 0.13 | 1.18 | 0.24 | 0.92 | 1.43 |
| Frequency of watching television | Not at all |  |  |  |  |  |  |
|  | Less than once a week | 0.87 | 0.18 | -0.70 | 0.49 | 0.58 | 1.29 |
|  | At least once a week | 1.03 | 0.14 | 0.19 | 0.85 | 0.78 | 1.35 |
| Distance to health facility | Big problem |  |  |  |  |  |  |
|  | Not a big problem | 0.96 | 0.11 | -0.37 | 0.71 | 0.77 | 1.20 |
| Place of residence | Urban |  |  |  |  |  |  |
|  | Rural | 0.93 | 0.11 | -0.59 | 0.55 | 0.75 | 1.17 |
|  | _cons | 0.25 | 0.13 | -2.71 | 0.01 | 0.09 | 0.68 |
| Cluster | v001 |  |  |  |  |  |  |
|  | var(_cons) | 5.27E-35 | 1.14E-18 |  |  | . | . |
