# Supplementary material for: Wealth-based inequity in full child vaccination coverage: An experience from Mali, Bangladesh, and South Africa. A multilevel poison regression
Source: PLoS One. 2023 Dec 20;18(12):e0293522. doi: 10.1371/journal.pone.0293522 (PMC10732374; doi:10.1371/journal.pone.0293522)
Supplement: Attachment — Submitted filename: Response letter_PLOS ONE.pdf [file pone.0293522.s003.pdf]

Ref: Submission ID PONE-D-22-32266R1

Title: Wealth-based inequity in full child vaccination coverage: An experience from Mali, Bangladesh, and South Africa. A multilevel poison regression.

Response to editor and reviewers' comments

Response to editor comments

Dear Editor,

Thank you very much for taking the time to review our submission, and for allowing us to revise our manuscript. We revised our manuscript based on the comments raised by the editor and the reviewers. We found all of them to be relevant to improve the article; accordingly, we prepared three files for submission including the clean manuscript, the manuscript with track changes, and this rebuttal letter. Please kindly find the point-by-point response below.

1. Improve the Introduction and Discussion sections based on the most recent article published on this topic considering three countries Bangladesh, Mali, and South Africa.

> Response: We considered your suggestion carefully to revise the introduction and discussion sections and updated them with available recent evidence in the area.

2. Revise the Methods section as per the reviewer's comment.

> Response: Thank you very much. We have now modified the methods section according to the reviewer's comment.

Response to reviewer two

Dear reviewer,

Thank you very much for spending your precious time revising our manuscript and providing your valuable comments. The suggestions you made are important to improve our work. Accordingly, we have used all your comments and made changes to the revised copy of the manuscript.

1. Please, define the multilevel-Poison regression model in the methods section. Check the Multi-collinearity among the explanatory variables.

> Response: Thank you very much again. We described multilevel-poison regression in the last paragraph of the statistical analysis section. Regarding the multi-collinearity, we have checked for the possible correlation between the predictor variables during the data analysis, and no correlations were detected among them.

Response to reviewer three

Dear reviewer,

Thank you very much for taking your valuable time to review our manuscript. We appreciate all your efforts to the improvement of our article. All the comments and suggestions you made were very relevant, and we accepted all of them and revised the manuscript's main document accordingly. Below, we explained all of your concerns in a point-by-point fashion as follows:

The study is well structured and provides important insights into the factors affecting child vaccination in different economic settings. However, there are a few areas where the authors can consider making improvements to enhance the clarity and relevance of their work.

1. Abstract clarity and precision: The abstract is a crucial section that provides a snapshot of the study's objectives, methods, results, and conclusions. In this case, the abstract seems to be a bit dense and could benefit from being more concise and precise. Focus on highlighting the main findings, methods, and implications of the study.

> **Response:** We agree with your comment, and we have modified the abstract section making it more precise and focused accordingly.

2. Data Source Details: in the data source/extraction" section, consider providing more details about the Demographic and Health Survey (DHS) database, such as its purpose, methodology, and coverage. This would help readers understand the source of the data better.

> **Response:** thank you very much again. We have now included the purpose, methodology, and coverage of the DHS program in the data source/extraction section of the revised manuscript.

3. Equity Analysis Visualizations: The concentration curves and index values are important visualizations to illustrate wealth-based inequities. Consider including these visualizations in the main body of the paper (not just in the reviewer's comments) to help readers better understand the disparities.

> **Response:** Including the illustrations/figures will indeed help the readers a lot to capture the information provided. We have included the concentration curve indicating the equity differences in full child vaccination; and an illustration of the concentration index of equity differences stratified by place of residence (urban-rural) in Figure 2, and 3 respectively, in wealth wealth-based inequality sub-section of the result section of the main manuscript.

4. Discussion: In the discussion section, consider placing the study's findings in the broader global context. How do the identified disparities and predictors align with trends observed in other low- and middle-income countries? Discuss any potential policy implications that could be generalized to other regions with similar contexts.

> **Response:** We accepted your comment fully. We have now modified it by narrating the scenario with more other LMIC countries, and we have suggested the possible implication for action in paragraph four of the discussion section.

5. Limitations and Future Research: While you've discussed the strength of the study, also include a section on limitations. Address any potential biases in the data, assumptions made during analysis, and any limitations of the chosen methodology. Suggest areas for future research, such as exploring other factors that might contribute to vaccination disparities.

> Response: We appreciate the suggestion. In the revised copy of our manuscript, we have discussed the possible limitations of our study: the secondary nature of the data does not allow recruiting many variables of interest, recall bias might have been introduced and the possibilities of unseen variable bias also suggested areas for future researchers in the strength and limitation section of the revised manuscript.

6. Clarity and Flow: Proofread the manuscript to ensure smooth readability and clarity. Make sure that each section flows logically into the next and that the writing is concise and precise.

> **Response:** Dear reviewer, thanks for your suggestion. We have proofread the whole manuscript and made necessary edits throughout the document.
